# A diagnostic algorithm for the surveillance of deep surgical site infections after colorectal surgery

**DOI:** 10.1017/ice.2019.36

**Published:** 2019-05

**Authors:** Tessa Mulder, Marjolein F.Q. Kluytmans-van den Bergh, Maaike S.M. van Mourik, Jannie Romme, Rogier M.P.H. Crolla, Marc J.M. Bonten, Jan A.J.W. Kluytmans

**Affiliations:** 1 Julius Center for Health Sciences and Primary Care, University Medical Center Utrecht, University of Utrecht, Utrecht, The Netherlands; 2 Amphia Academy Infectious Disease Foundation, Amphia Hospital, Breda, The Netherlands; 3 Department of Infection Control, Amphia Hospital, Breda, The Netherlands; 4 Department of Medical Microbiology, University Medical Center Utrecht, University of Utrecht, Utrecht, The Netherlands; 5 Department of Surgery, Amphia Hospital, Breda, The Netherlands

## Abstract

**Objective:**

Surveillance of surgical site infections (SSIs) is important for infection control and is usually performed through retrospective manual chart review. The aim of this study was to develop an algorithm for the surveillance of deep SSIs based on clinical variables to enhance efficiency of surveillance.

**Design:**

Retrospective cohort study (2012–2015).

**Setting:**

A Dutch teaching hospital.

**Participants:**

We included all consecutive patients who underwent colorectal surgery excluding those with contaminated wounds at the time of surgery. All patients were evaluated for deep SSIs through manual chart review, using the Centers for Disease Control and Prevention (CDC) criteria as the reference standard.

**Analysis:**

We used logistic regression modeling to identify predictors that contributed to the estimation of diagnostic probability. Bootstrapping was applied to increase generalizability, followed by assessment of statistical performance and clinical implications.

**Results:**

In total, 1,606 patients were included, of whom 129 (8.0%) acquired a deep SSI. The final model included postoperative length of stay, wound class, readmission, reoperation, and 30-day mortality. The model achieved 68.7% specificity and 98.5% sensitivity and an area under the receiver operator characteristic (ROC) curve (AUC) of 0.950 (95% CI, 0.932–0.969). Positive and negative predictive values were 21.5% and 99.8%, respectively. Applying the algorithm resulted in a 63.4% reduction in the number of records requiring full manual review (from 1,606 to 590).

**Conclusions:**

This 5-parameter model identified 98.5% of patients with a deep SSI. The model can be used to develop semiautomatic surveillance of deep SSIs after colorectal surgery, which may further improve efficiency and quality of SSI surveillance.

Surgical site infections (SSIs) are among the most common healthcare-associated infections (HAIs) in surgical patients.^1^ SSIs are associated with a substantial clinical and financial burden due to their negative impact on patient health and the increased costs associated with treatment and extended hospitalization.^2^^,^^3^ On average, these infections occur in 2%–4% of surgical patients, but the infection rates and severity vary across surgical procedures.^4^^,^^5^ Colorectal surgery is associated with the highest risk of infection, which ranges from 15% to 30% of these patients.^6^

The incidence of SSIs and other HAIs are monitored in infection surveillance programs. These programs routinely collect data on HAI rates, which are used as reference data for hospitals and healthcare providers.^7^ Data can be applied to evaluate the quality of care, to identify where improvements are needed, and to support and facilitate implementation of new preventive measures. As a result, infection surveillance can be used to reduce SSI-related morbidity and costs. To ensure adequate and reliable surveillance, uniform ascertainment of infection is crucial. International definitions were therefore developed to provide a standardized approach to diagnosing and reporting SSIs.^8^ SSI surveillance is traditionally performed by extensive manual review of medical records. Consequently, surveillance is labor intensive and time-consuming. As the number of surgical procedures continues to rise, the total time spent on surveillance likely will increase as well.^9^

The objective of this study was to develop an algorithm for the surveillance of deep SSIs after colorectal surgery to reduce the number of medical records that require full manual review.

## Methods

### Study design

This retrospective cohort study was conducted in the Amphia Hospital (Breda, The Netherlands), a teaching hospital that participates in the national HAI surveillance program. Medical records of all patients who undergo colorectal surgery are manually reviewed by trained infection control practitioners for the identification of deep SSI. Deep SSIs are defined according to the Centers for Disease Control and Prevention (CDC) criteria and comprise deep incisional and organ/space infections that manifest within 30 days after surgery.^8^ Postoperative complications that developed after discharged were either reported when the patient was referred back to the hospital or during a postoperative outpatient clinic visit ∼30 days after surgery. For the present analysis, we used surveillance data from January 2012 through December 2015. Infection surveillance was performed by the same infection control practitioners throughout the entire study period. A multidisciplinary group of infection control practitioners, surgeons, and clinical microbiologists discussed cases to reach consensus on the diagnosis when necessary.

Patients who were categorized as having a contaminated wound at the time of the surgical procedure (ie, wound class 4) were excluded because these wounds were already infected at the time of the surgical procedure. Demographic and clinical patient data and data on the surgical procedure were collected by manual chart review, except for data on postoperative use of antibiotics and diagnostic radiological procedures, which were automatically extracted.

We designed the analysis by following the TRIPOD statement for prediction modeling.^10^ Candidate model predictors were selected based on literature and included known risk factors or outcomes for deep SSI.^3^^,^^11^^,^^12^^,^^13^ One predictor was selected for every 10 events. The administration of preoperative oral and perioperative intravenous antibiotic prophylaxis, American Society of Anesthesiologists (ASA) score classification, wound class, level of emergency, blood loss during the procedure, surgical technique were selected as preoperative and operative candidate predictors. Preoperative oral antibiotic prophylaxis comprised a 3-day course of colistin and tobramycin. Perioperative intravenous prophylaxis was administered according to the national guideline.^14^ Surgical technique was categorized into open, laparoscopic, and robotic laparoscopic procedures. Laparoscopic procedures that were converted to open were categorized as open procedures. The postoperative predictors length of stay, hospital readmission, reoperation, mortality, and in-hospital antibiotic use and abdominal radiologic procedures were assessed 30 days after the primary surgical procedure. Data on 30-day mortality were collected from the hospital database.

### Statistical analysis

Univariable associations between baseline characteristics and candidate predictors and deep SSI were estimated using the Student *t* test or the Mann-Whitney *U* test for continuous variables, and the Fisher exact or χ^2^ test for categorical variables. We analyzed missing data by comparing patients with complete data with patients who had 1 or more missing values in the outcome or in the model predictors. Missing data were subsequently imputed using multiple imputation by chained equations (MICE), and 10 datasets were created.^15^ Predictive mean matching and logistic regression were used as imputation techniques for continuous and binary variables, respectively. Rubin’s rule was applied to calculate pooled results.^16^ We built the model using multivariable logistic regression analysis with backward selection. The Akaike information criterion (AIC) was used to determine whether the model fit could be improved by deleting predictors. Predictors were deleted until the AIC could not be further reduced. The final model was validated internally with bootstrapping to correct for optimism (500 samples).^17^

We determined discriminatory power and calibration to evaluate the statistical performance of the model.^18,^ ^19^ Discrimination was defined as the area under the receiver operating characteristic (ROC) curve (AUC). Calibration of the model was tested by plotting the predicted probabilities against the observed outcomes in the cohort. The final step was to evaluate the clinical applicability of the model. A predicted probability threshold was selected corresponding to an excellent sensitivity and an acceptable specificity to ensure that the vast majority of cases would be detected. When the individual predicted probabilities exceeded the predefined threshold, the model identified the medical record as having had a high probability for deep SSI, and the medical record was kept for full manual review. Not exceeding the threshold led to immediate classification of the record as ‘no deep SSI.’ At the threshold, the associated sensitivity, specificity, positive predictive value, and negative predictive value were calculated. As an exploratory analysis, we also assessed the diagnostic performance of the strongest predictor. *P* < .05 (2-sided) was considered statistically significant. All statistical analyses were performed using R version 1.0.143.^20^

## Results

In total, 1,717 patients underwent colorectal surgery in the study period, of whom 111 (6.5%) with a contaminated wound (wound class 4) were excluded. Of the 1,606 remaining patients, 129 patients acquired a deep SSI (8.0%). Baseline characteristics are presented in Table 1. The median age was 68 years and 55.7% of patients were male. Compared with patients without SSI, patients who acquired an SSI less frequently received prophylactic oral antibiotics before surgery (44.2% vs 60.3%); more frequently had ASA scores of ≥2 (43.1% vs 28.7%); and had increased risks of reoperation (79.1% vs 6.1%), readmission (24.8% vs 8.9%), and death (8.5% vs 2.3%). Baseline characteristics for patients with complete data and for patients with 1 or more missing values are shown in Supplementary Table 1 online. Moreover, 109 (6.8%) were missing data for at least 1 of the covariables (6.8%) Patients with complete data differed significantly from those with missing data, which supported the use of multiple imputation to reduce the risk of bias due to missing data.

**Table 1.** Baseline Characteristics (Before Imputation)

Variable	No SSI (n=1,477), No. (%)^a^	SSI (n=129), No. (%)^a^	*P* Value^b^
**Patient characteristics**			
Age, y (IQR)	68 (60–76)	67 (60–75)	.583
Male	823 (55.7)	72 (55.8)	.912
BMI, kg/m^3^ (IQR)^c^	25 (23–28)	25 (23–28)	.076
Preoperative oral antibiotic prophylaxis	891 (60.3)	57 (44.2)	<.001
ASA classification >2^c^	400 (28.7)	53 (43.1)	<.001
**Wound class**			
Clean contaminated (class 2)	1,355 (91.7)	112 (86.8)	.082
Contaminated (class 3)	122 (8.3)	17 (13.2)	
Blood loss (mL)	3 (1–56)	24 (1–60)	.175
Normothermia	1,049 (91.9)	95 (91.3)	.981
Implant of nonhuman tissue	4 (0.3)	1 (0.8)	.871
Perioperative antibiotic prophylaxis^c^	1,402 (95.7)	119 (93.7)	.411
Colorectal malignancy	1,112 (75.3)	90 (69.8)	.201
Surgery in preceding year	169 (11.4)	13 (10.1)	.746
Experienced surgeon^d^	1,150 (77.9)	88 (68.2)	.017
Multiple surgical procedures^e^	282 (19.1)	29 (22.5)	.414
**Surgical approach**^c^			
Open	729 (49.6)	80 (62.0)	.022
Conventional laparoscopic	530 (36.1)	33 (25.6)	
Robotic laparoscopic	211 (14.4)	16 (12.4)	
Duration of surgery >75th percentile^f^	358 (24.2)	35 (27.1)	.531
**Level of emergency**			
Acute	52 (3.5)	8 (6.2)	.194
Elective	1,425 (96.5)	121 (93.8)	
**Postoperative course, 30 d after the procedure**^g^			
Reoperation	90 (6.1)	102 (79.1)	<.001
Readmission	132 (8.9)	32 (24.8)	<.001
ICU admission	127 (8.6)	67 (51.9)	<.001
Death	34 (2.3)	11 (8.5)	<.001
Length of stay,^d^	7 (5-11)	24 (13-38)	<.001
Abdominal radiological examination^h^	122 (8.3)	45 (34.9)	<.001
Antibiotic use^d^	241 (16.3)	70 (54.3)	<.001

Note. ICU, intensive care unit; IQR, interquartile range; SSI, surgical site infection; BMI, body mass index.

a Data are presented as no. (%) or median (IQR).

b *P* values are the estimated univariable associations between the variable and deep SSI.

c % missing data: ASA classification, 5.5%; perioperative antibiotic prophylaxis, 0.85%; surgical approach, 0.44%; BMI, 1.75%.

d Performed at least 25 colorectal surgical procedures in 1 year.

e Multiple surgical incisions during the same surgical procedure, excludes creation of ostomy.

f 75^th^ percentiles of duration of surgery accounting for the n type of resection and for the surgical approach, according to PREZIES reference values.^21^

g Evaluation of the postoperative outcomes occurred 30 d after the index procedure.

h Starting 48 h after the primary procedure.

**Fig. 1.**
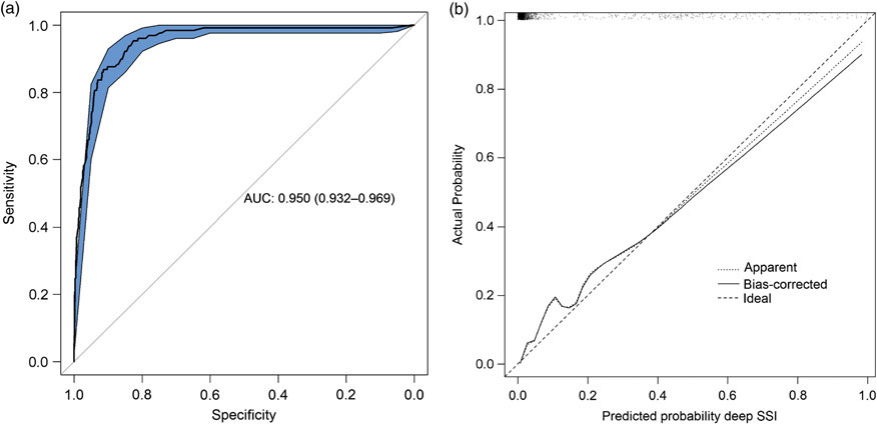
Statistical model performance. A. ROC curve with discriminatory power expressed as AUC (AUC, 0.950; 95% CI, 0.932–0.969). B. Calibration plot of the model. Calibration refers to the correspondence between the probability of SSI predicted by the model and the actual probability of infection. The diagonal line represents perfect (ideal) calibration; the dotted line represents the actual calibration; and the black line represents calibration after bootstrapping. The slope of the linear predictor was 0.978, indicating slight overprediction before bootstrapping. Note. ROC, receiver operating characteristic; AUC, area under the curve; CI, confidence interval; SSI, surgical site infection.

**Fig. 2.**
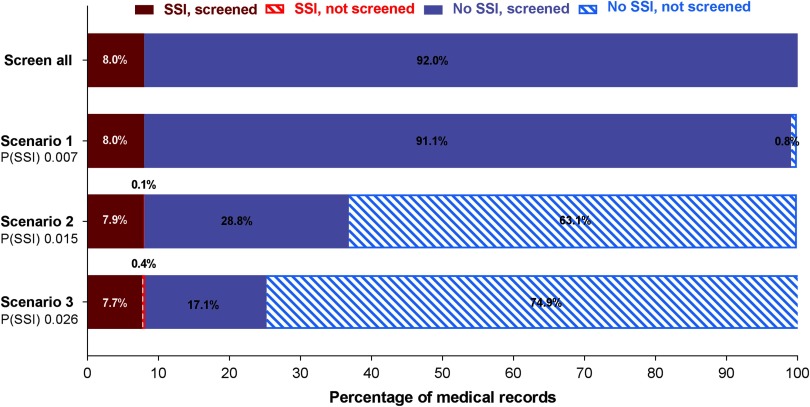
Clinical applicability of the prediction model. Manual screening of all files is compared with screening a subset of files that are preselected by the prediction model. Three scenarios with different cutoffs in predicted probability for SSI are presented. When the predicted probability for a patient exceeds the cutoff value, the patient file will be identified as a possible SSI and will be retained for manual review. If the threshold is not exceeded, the patient file will be discarded immediately. The solid bars represent the files that are manually screened; the striped bars represent the files that are discarded. When the predicted probability cut off increases, the number of files that need to be reviewed manually decreases. The number of missed SSI cases (ie, false negatives) will increase. Note. P(SSI), predicted probability for surgical site infection.

The final diagnostic model, including bias-corrected estimates, is shown in Table 2. The predictors retained in the model after backward selection were wound class, reoperation, readmission, length of stay, and death. The prediction rule is illustrated in Supplementary Fig. 1 online. The discriminatory power of the final model was 0.950 (95% CI, 0.932–0.969) (Fig. 1A), with good calibration (Fig. 1B). The calibration slope that was used to shrink the estimates was 0.978, indicating slight overprediction of the model before bootstrapping. To assess the clinical applicability of the model (Fig. 2), we compared complete chart review with the results produced by the final model when we used 3 different predicted probability thresholds.

**Table 2.** Final Model for the Prediction of Deep SSI^a^

Predictor	Model Estimates	
	OR	95% CI
Wound class		
Clean-contaminated (class 2)	Reference	Reference
Contaminated (class 3)	2.25	0.95–4.88
Hospital readmission	3.97	1.92–7.72
Reoperation	26.20	13.95–14.43
Postoperative length of stay, d	1.07	1.05–1.09
Death	3.09	1.23–7.57

Note. CI, confidence interval; OR, odds ratio; SSI, surgical site infection.

a Final logistic regression model after backward selection on Akaike information criterion Intercept: −5.234. ORs and CIs were corrected for optimism by bootstrapping (2,000 samples). The following predictors were not retained in the model: ASA classification, level of emergency, preoperative oral antibiotic prophylaxis, blood loss during surgery, surgical approach, administration of antibiotics and the requests for radiology of the abdomen. All patients (n= 1,616) had complete data for all 5 parameters.

At the predicted probability threshold of 0.015, the model had a 98.5% sensitivity, 68.7% specificity, 21.5% positive predictive value, and 99.8% negative predictive value for predicting deep SSI (Table 3). The number of medical records that required manual review was reduced from 100% to 36.7% (from 1,606 to 590 records). Finally, the diagnostic performance of the strongest predictor (reoperation) was evaluated. Reoperation was strongly associated with deep SSI and had good discriminatory power (AUC, 0.865; 95% CI, 0.829–0.901), with 79.1% sensitivity, 93.7% specificity, 53.1% positive predictive value, and 98.1% negative predictive value. With this single predictor, the number of charts to review manually was reduced to 11.9% (from 1,606 to 192 records).

**Table 3.** Contingency Table for the Prediction of Deep Surgical Site Infection (SSI)

Predicted Probability	Deep SSI			
	Yes	No	Total	
*P*(SSI) ≥ .015	127	463	590	PPV, 21.5% (95% CI, 20.2–22.9)
*P*(SSI) < .015	2	1,014	1,016	NPV, 99.8% (95% CI, 99.2–99.9)
Total	129	1,477	1,606	
	Sensitivity, 98.5% (95% CI, 94.5–99.8)	Specificity, 68.7% (95% CI, 66.2–71.0)		

Note. CI, confidence interval; NPV, negative predictive value; P(SSI), probability of surgical site infection; PPV, positive predictive value.

## Discussion

We developed a 5-parameter diagnostic model that was able to identify 98.5% of all deep SSIs with a 99.8% negative predictive value. Use of the model could reduce the number of medical records that required full manual review from 1,606 (100%) to 590 (36.7%), at the cost of 2 missed deep SSIs (1.6%). Increasing the predicted probability threshold would allow for further reduction of workload but would also increase the likelihood of missed SSIs. Using only reoperation to detect deep SSI, the number of medical records for complete manual review was substantially reduced, although this method is associated with a higher false-negative rate and lower discriminatory power compared to the full model. Nevertheless, we suggest that reoperation should be included as a model parameter when new algorithms are developed for colorectal SSI surveillance. We set the model threshold at an excellent sensitivity so that it could detect a high percentage of patients with SSI. The trade-off for this high sensitivity was a relatively high rate of false positives due to a moderate specificity, which we accepted because the model would be used to identify the patients with a high probability of SSI whose records would then be reviewed. Thus, the lower specificity would not affect case finding. The moderate false-positive rate decreases the efficiency of the model. However, SSI surveillance that uses the model to identify patients whose records need to be reviewed will be more efficient than reviewing every patient’s record. Because this model has a high sensitivity, it could be used for quality improvement purposes and for benchmarking.

Previous studies of drain-related meningitis,^22^ bloodstream infections,^23^ and SSI after orthopedic procedures^24^ found that algorithms performed well compared with manual surveillance. For the surveillance of SSIs after gastrointestinal surgery, algorithms have been developed using several approaches, such as Bayesian network modelling (AUC, 0.89),^25^ logistic regression modeling (AUC, 0.89),^26^ or machine learning (AUC, 0.82).^27^

We made several efforts to secure generalizability and validity of our findings. We aimed to take an objective approach in model development by selecting predictors on theoretical grounds exclusively and by performing backward selection using the statistical model fit. Subsequently, the model was internally validated and, as such, we attempted to reduce the risk of overprediction. We obtained complete data on the outcome with the reference standard, and we did not change the definition of SSI the during the study period, which prevents selection bias due to partial verification.

Several limitations should be addressed. The use of routine care data as well as retrospective data collection is inevitably associated with missing information. Proper handling of missing data reduces the risk of bias and improves precision. We applied multiple imputation (MICE) to handle the missing data, which has been demonstrated previously to improve the performance of automated detection of SSI.^27^ When this model is used in clinical practice, missing data cannot be dealt with using the same method, which is an issue with applying prediction models in general. In the case of missing values, the model cannot calculate a predicted probability. Using backward selection in algorithm development, the number of parameters is reduced which also limits the amount of information needed for adequate diagnostic performance. As such, the clinical applicability will be enhanced because the risk of missing values is reduced. We had no missing data in any of the model covariables; thus, we expect that missing data will not be an important issue when the algorithm is applied in clinical practice.

Another limitation is that the model was developed on data derived from a single hospital. The parameters in the final model should be available in most medical records. Hospitals wishing to validate the algorithm in their colorectal surgical population should therefore ensure that all of the parameters are available electronically. Also, external validation is essential before the model can be applied in practice. Ideally, data derived from multiple hospitals are used to confirm model performance in other settings and future studies may also investigate less data-driven methods of model development for semiautomated surveillance.

In conclusion, we developed a 5-parameter diagnostic model that identified 98.5% of the patients who acquired a deep SSI and reduced the number of medical records requiring complete manual screening by 63.4%. These results can be used to develop semiautomatic surveillance of deep SSIs after colorectal surgery.

## Supplementary material

For supplementary material accompanying this paper visit https://doi.org/10.1017/ice.2019.36. click here to view supplementary material
