# Supplementary material for: A diagnostic algorithm for the surveillance of deep surgical site infections after colorectal surgery
Source: Infect Control Hosp Epidemiol. 2019 May;40(5):574–8. doi: 10.1017/ice.2019.36 (PMC6536899; doi:10.1017/ice.2019.36)
Supplement: Supplementary file 1 [file S0899823X19000369supp001.docx]

**Supplementary material**

| **Supplementary Table 1** Distribution of variables among patients without and with missing values | | | |
| --- | --- | --- | --- |
| **Variable** | **No missings**  **n = 1,497 (93.2)** | **≥ 1 missing**  **n = 109 (6.8)** | ***P*-value** |
| **Patient characteristics** | | | |
| Age (years) | 68 (60-67) | 68 (59-77) | 0.931 |
| Male sex | 839 (56) | 56 (51.4) | 0.397 |
| BMI (kg/m^3^) | 25 (23-28) | 25 (22-29) | 0.976 |
| Preoperative oral antibiotic prophylaxis | 909 (60.7) | 28 (25.7) | <0.001 |
| ASA classification >2 | 438 (29.3) | 15 (71.4) | <0.001 |
| **Operative characteristics** | | | |
| Wound class |  |  |  |
| Clean contaminated | 1,375 (91.9) | 92 (84.4) | 0.013 |
| Contaminated | 122 (8.1) | 17 (15.6) |  |
| Operative duration > 75^th^ percentile | 379 (25.3) | 14 (12.8) | 0.005 |
| Normothermia during surgery | 1,069 (91.8) | 75 (93.8) | 0.675 |
| Blood loss (mL) | 3 (1-56) | 24 (1-60) | 0.447 |
| Implant of non-human tissue | 5 (0.3) | 0 | 1.000 |
| Perioperative antibiotic prophylaxis | 1,436 (95.9) | 85 (89.5) | 0.007 |
| Colorectal malignancy | 1,130 (75.5) | 72 (66.1) | 0.038 |
| Surgery in preceding year | 176 (19.2) | 6 (5.5) | 0.067 |
| Experienced surgeon ^a^ | 1,180 (78.8) | 58 (53.2) | <0.001 |
| Multiple surgical procedures ^b^ | 287 (19.2) | 24 (22.0) | 0.548 |
| Surgical approach |  |  |  |
| Open ^c^ | 729 (48.7) | 80 (78.4) | <0.001 |
| Conventional laparoscopic | 548 (36.6) | 15 (14.7) |  |
| Robotic laparoscopic | 220 (14.7) | 7 (6.9) |  |
| Level of emergency |  |  |  |
| Acute | 48 (3.2) | 12 (11.0) | <0.001 |
| Elective | 1449 (96.8) | 97 (89.0) |  |
| **Postoperative course, 30 days after the procedure** | | | |
| Reoperation | 119 (11.1) | 73 (13.7) | 0.157 |
| Readmission | 106 (9.9) | 58 (10.9) | 0.603 |
| ICU admission | 112 (10.4) | 82 (15.4) | 0.006 |
| Death | 20 (1.9) | 25 (4.7) | 0.002 |
| Length of stay in days, *median (IQR)* | 6 (4-10) | 7 (5-12) | <0.001 |
| Abdominal radiological examination ^d^ | 155 (14.5) | 12 (2.5) | <0.001 |
| Prescription of antibiotics ^d^ | 290 (27.1) | 21 (32.2) | 0.436 |
| **Outcome** |  |  |  |
| Deep SSI | 121 (8.1) | 8 (7.3) | 0.926 |
| Comparison of complete cases to patients with one or more missing values. Complete cases were defined as absence of missing values in any of the covariables or in the outcome of the multivariable model. Data are presented as n (%) or median (interquartile range).  ^a^ Performed at least 25 colorectal surgical procedures in one year.  ^b^ Multiple surgical incisions during the same surgical procedure, excludes creation of ostomy  ^c^ 75^th^ percentiles of duration of surgery accounting for the n type of resection and for the surgical approach, according to PREZIES reference values. ^27^  ^d^ Starting 48 hours after the primary procedure  Abbreviations: ICU: Intensive Care Unit; IQR: Interquartile Range; SSI: Surgical Site Infection | | | |


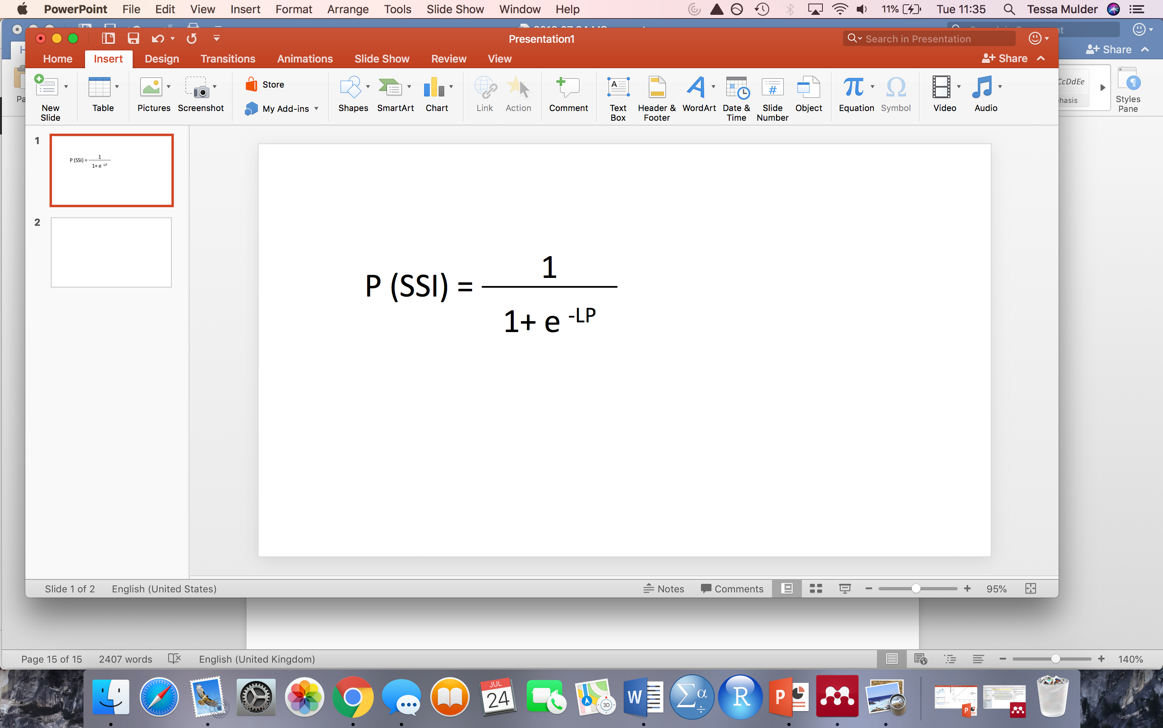


LP = -5.234 + 0.890* contaminated wound (class 3) + 3.037 * reoperation + 1.489 * readmission + 0.085 * number of postoperative days admitted to the hospital + 1.127 * death

**Supplementary figure 1** Prediction rule for deep surgical site infections

Abbreviations: LP: linear predictor; P(SSI): predicted probability for surgical site infection
